# Comparison of oxidative stress among migraineurs, tension-type headache subjects, and a control group

**DOI:** 10.4103/0972-2327.56316

**Published:** 2009

**Authors:** Ravi Gupta, Rahul Pathak, Manjeet Singh Bhatia, Basu Deb Banerjee

**Affiliations:** Departments of Psychiatry, University College of Medical Sciences and GTB Hospital, Dilshad Garden, Delhi-110 095, India; 1 Departments of Biochemistry, University College of Medical Sciences and GTB Hospital, Dilshad Garden, Delhi-110 095, India

**Keywords:** Migraine, tension type headache, oxidative stress, ferric reducing activity of plasma, malondialdehyde

## Abstract

**Background:**

A primary headache, particularly migraine, is associated with oxidative stress during the attack. However, data regarding the interictal state in migraineurs and in those with tension-type headache (TTH) is limited.

**Objectives:**

(1) To assess the oxidative stress in migraineurs and TTH subjects in between the episodes and (2) to see if there is a difference in the degree of oxidative stress in the different subtypes of migraine and TTH.

**Materials and Methods:**

Fifty migraineurs, 50 patients with TTH, and 50 control subjects were included in this study after screening for the exclusion criteria. Diagnosis of headache was made according to the International Classification of Headache Disorders (ICHD)-2 criteria. A venous blood sample was collected from the antecubital vein at least 3 days after the last attack of headache. The sample was centrifuged immediately and the plasma was stored at –70°C. The ferric reducing activity of plasma (FRAP) and the malondialdehyde (MDA) levels were assessed using colorimetric methods. Statistical analysis was done with the help of SPSS for Windows, v 11.0. One way ANOVA with post hoc Tukey test, independent sample t test, univariate regression, and multivariate regression analysis were done as indicated.

**Results:**

Migraineurs had higher values of MDA and FRAP than the subjects in the other two groups (*P*<0.001). No difference was observed between the TTH group and the control group. FRAP levels were significantly higher in subjects who had mixed migraine (migraine with aura and without aura) as compared to those with only migraine without aura (mean difference 196.1; 95% CI = 27.3 to 364.9; *P* = 0.01). Similarly, oxidative stress was significantly higher in patients with episodic TTH as compared to those with chronic TTH (FRAP t = 3.16; *P* = 0.003 and MDA t = 2.75; *P* = 0.008).

**Conclusions:**

This study suggests that oxidative stress continues even between headache episodes in migraineurs but not in those with TTH. This could probably be consequent to the different pathophysiological mechanisms of TTH and migraine.

## Introduction

Primary headaches, e.g., migraine and tension-type headache (TTH), are associated with biochemical abnormalities of the brain. Some of these aberrations predispose a person to headache, while others are pathophysiologically associated with the headache; however, a cause-and-effect relationship is yet to be established. The presence of mitochondrial abnormalities in migraineurs has been known since a long time, and impaired energy phosphate metabolism has been described during the initial phase of migraine.[1] This leads to anaerobic metabolism, which makes the cell prone to oxidative stress.[2] Besides this, other mechanisms also play a part in the development of oxidative stress in headache patients, viz., release of proinflammatory cytokines during headache;[3,4] formation of nitric oxide (NO) in the endothelium and perivascular spaces during a migraine attack[5] which, being an unstable molecule, rapidly converts to peroxynitrite;[6] NO-mediated release of arachidonic acid metabolites,[7,8] which can invoke oxidative stress;[9] and, lastly, associated psychological stress, which silently invokes oxidative damage to the body.[10,11]

Although studies on oxidative stress in primary headache are limited, available literature has described the increment in the markers of oxidative stress among primary headache patients. Superoxide dismutase (SOD) is a protective enzyme that neutralizes superoxide. Its level was lower among migraineurs than in a control population.[12] Furthermore, it was lowest in subjects suffering migraine with aura followed by patients having migraine without aura, suggesting that the migraine subtype can have an effect on oxidant production. TTH subjects had lower levels than controls, but the difference was not significant.[12] The oxidant mediates lipid peroxidation inside our body,[13] and increased levels of lipid peroxides products have been reported in migraineurs during headache[14–16] but not when there is no headache.[16] Similarly, at least one study has suggested that aura does not affect the oxidant status of migraineurs.[14] Hence, the literature is contradictory, generally favoring the episodic occurrence of oxidative stress during migraine attacks, with biochemical quiescence in between episodes. Moreover, to the best of our knowledge, oxidative stress parameters have never been examined in TTH patients, except in one study.[12]

Hence, present study was planned to assess the total anti-oxidant activity of plasma as well as lipid peroxidation in migraineurs as well as TTH patients during inter-episodic period.

## Materials and Methods

We used convenience sampling to recruit 50 subjects suffering from migraine, 50 subjects of TTH, and 50 controls from the headache clinic of a teaching hospital. The study was approved by the institutional ethics committee. Informed consent was obtained from all the study subjects. Migraine and TTH were diagnosed according to the International Classification of Headache Disorders-2 (ICHD-2) criteria.[17] The migraineurs were divided into three subgroups: migraine with aura (MA), migraine without aura (MO), and mixed migraineurs, i.e, those having some attacks “with aura” and some “without aura” (MO-MA). Similarly, TTH group was divided into two subgroups: those with episodic TTH and those with chronic TTH.

The control group consisted of subjects who had never had recurrent primary headaches and in whom the family history was negative for primary headaches. All the participants belonged to the same ethnic group and had comparable socioeconomic status. None of the subjects were genetically related to any of the other subjects included in this study.

All the subjects were included only after they had been screened for the exclusion criteria. The exclusion criteria included the following: major neurological disorders (e.g., epilepsy, space-occupying lesions, or neurodegenerative disorders), chronic daily headaches (undiagnosed or mixed type), substance use disorders (except tobacco), a history of having taken prophylactic drugs for migraine or TTH for more than 3 weeks, comorbidities such as some other primary headache or a psychiatric disorder, and history of consumption of antioxidants or multivitamins for more than a week preceding the study.

The history of the headache was taken in detail; this was followed by the clinical examination and, wherever required, appropriate laboratory investigation to rule out secondary headache. Parallel information was also collected from a reliable family member regarding the patient's headaches. All the information was recorded on a semi-structured proforma.

### Measurement of oxidative stress

Fasting venous blood (3 ml) was collected in a sterile EDTA vaccutainer at least 3 days after the attack of headache; subjects were asked to take rest for at least 2 h before the blood was drawn. The sample was immediately centrifuged at 2000 rpm for 3 min and the plasma was transferred to another sterile vial and stored at –70°C till further use.

To assess the oxidative stress, plasma levels of malondialdehyde (MDA) and ferric reducing ability of plasma (FRAP) was measured according to the procedure described by Satoh[18] and Benzie and Strain,[19] respectively. For MDA measurement, 2.5 ml of 20% trichloroacetic acid was first added to 0.5 ml of serum. It was kept at room temperature for 10 min and then centrifuged at 3500 rpm for 10 min. The resultant supernatant was discarded and 2 ml of 0.05 M H_2_SO_4_ was poured into the test tube, which was then vortexed; following this the mixture was centrifuged again at 3500 rpm. After this, 2 ml of 0.05 M H_2_SO_4_ and 3 ml of thiobutyric acid were added. The tube was incubated in a water bath for 30 min at 60°C and then cooled under tap water. Following this, 4 ml of n-butanol was added to the test tube, which was vortexed for 5 min and then again centrifuged at 3000 rpm for 3 min. The upper organic layer was separated to read the absorbance.

For the FRAP measurement, fresh FRAP reagent was prepared by mixing 300 mM acetate buffer of pH 3.6 (prepared by adding 3.1 g sodium acetate trihydrate + 16 ml glacial acetic acid, with the final volume made up to 1 l with distilled water) with a mixture of 10 mM TPTZ (2,4,6-tripyridyltriazine) in 40 mM HCl and 20 mM FeCl_3_.6H_2_O in the ratio of 10:1:1. Then 3 ml of working FRAP reagent was mixed with 100 μl of the test sample in a test tube. It was vortex mixed and the absorbance was read against a reagent blank at a predetermined time (approximately 5 min) after sample reagent mixing.

Absorbance was read at 530 nm for MDA and at 593 nm for FRAP using a colorimeter. The results were plotted against the standard curves for each of them. The quantity of MDA was expressed in nmol/ml and for FRAP the values were expressed in μM.

### Statistical analysis

Analysis was done with the help of SPSS for Windows, v 11.0.0. The chi-square test was used to compare nonparametric variables. To compare the difference in the mean levels of MDA and FRAP between the three groups, one way ANOVA was used with post hoc Tukey wherever applicable. FRAP and MDA values in the two groups were compared with the help of the independent samples t test. Univariate regression analysis was done to find out the association between numerical variables.

## Results

The three groups were similar with respect to the average age of the subjects (migraineurs: 27.66 years; TTH group: 27.6 years; control group: 25.18 years) (F = 1.54; *P* = 0.21). In both the headache groups, females subjects formed the majority (82% among migraineurs and 100% in TTH group), whereas they were in a minority in the control group (25%) (X^2^ = 70.59, P<0.001). The BMI of the subjects was not significantly different among the three groups (migraineurs: 23.4; TTH: 22.7; control: 23.9) (*P*>0.05), and none of subjects fell into the category of “obese.”

Univariate regression analysis showed that MDA as well as FRAP levels were not influenced by age (R^2^ = 0.02 and R^2^ = 0.00, respectively). Similarly, gender did not have any effect on MDA (t = 0.5; *P* = 0.61) and FRAP (t = 1.44; *P* = 0.15) values.

**Figure 1:**
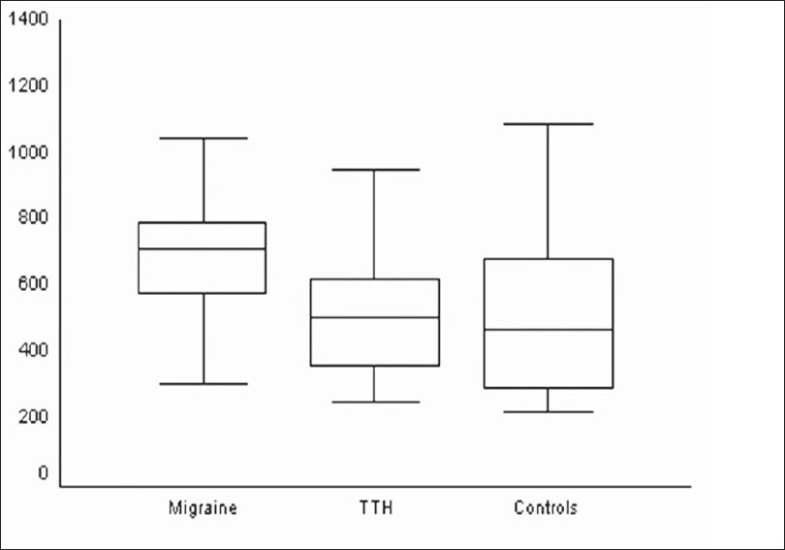
Comparison of plasma FRAP levels (μM) in the three groups (F = 12.68; P<0.001)

**Figure 2:**
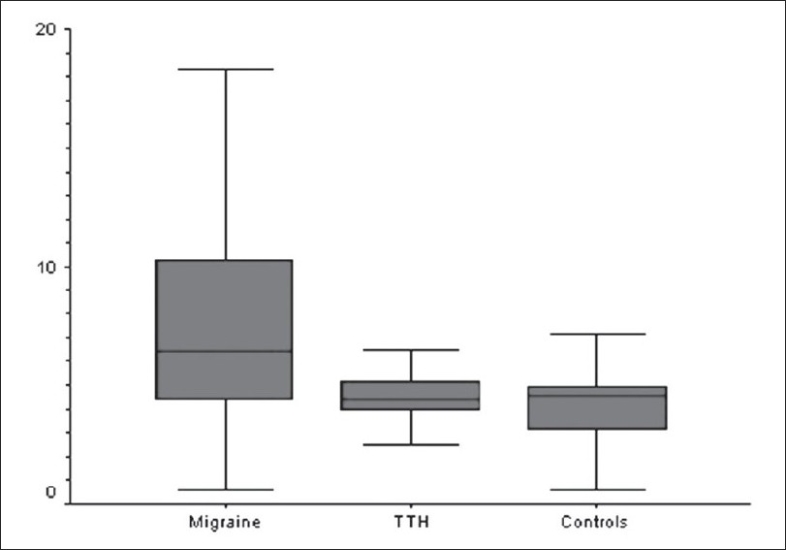
Comparison of plasma MDA (nmol/ml) levels in the three diagnostic groups (F = 20.36; P<0.001)

The migraine, TTH, and control groups were significantly different on the measures of oxidative stress. The highest values of MDA and FRAP were observed among migraineurs [Figures 1 and 2]. The post hoc Tukey test shows that migraineurs were significantly different from the other two groups with respect to both MDA (*P*<0.001) and FRAP (*P*<0.001). However, no significant difference was observed between the control group and the TTH group.

**Figure 3:**
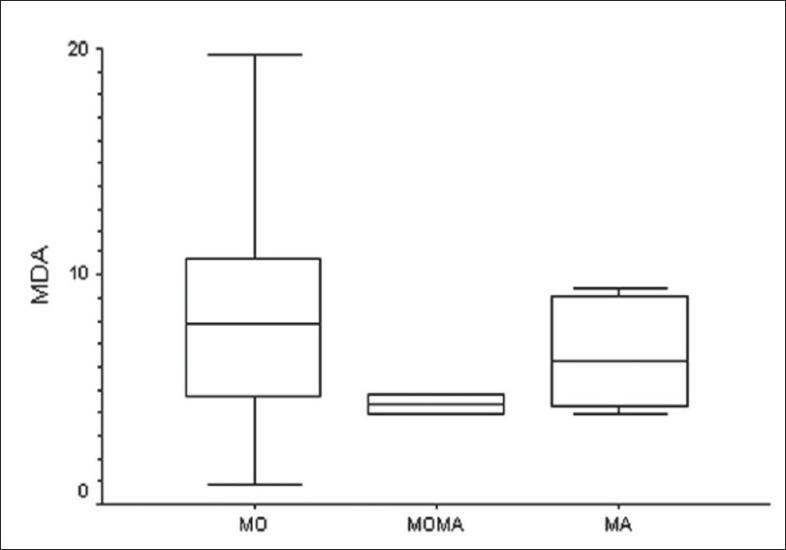
Comparison of plasma MDA levels (nmol/ml) in the three migraine subtypes

**Figure 4:**
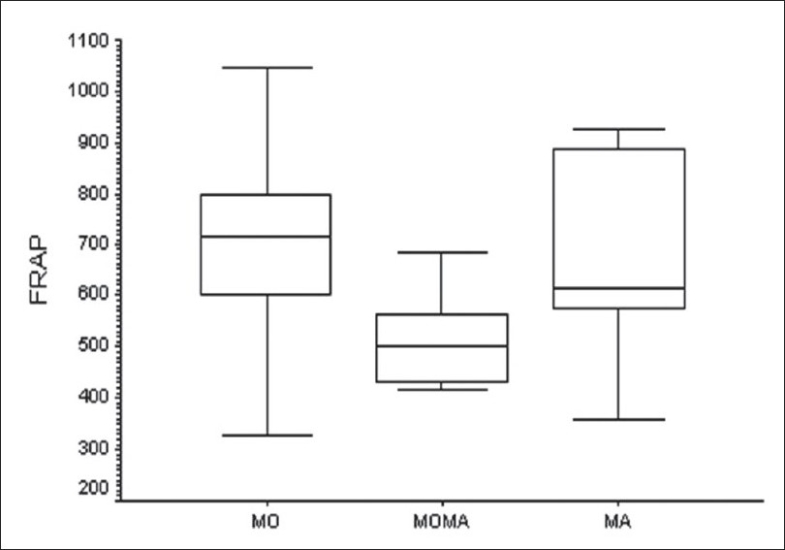
Comparison of plasma FRAP values (μM) among the three migraine subgroups

When the different subgroups among the migraineurs was examined, it was found that FRAP, but not the MDA levels, was significantly different among these subgroups (F = 3.97; *P* = 0.02 and F = 2.84; *P* = 0.06, respectively). Post hoc Tukey test showed that FRAP levels were significantly different between the MO subgroup and the MO-MA subgroup (mean difference 196.1; 95% CI = 27.3 to 364.9; *P* = 0.01). However, the MA subgroup was not significantly different from the other two subgroups with respect to the FRAP levels [Figures 3–4].

**Figure 5:**
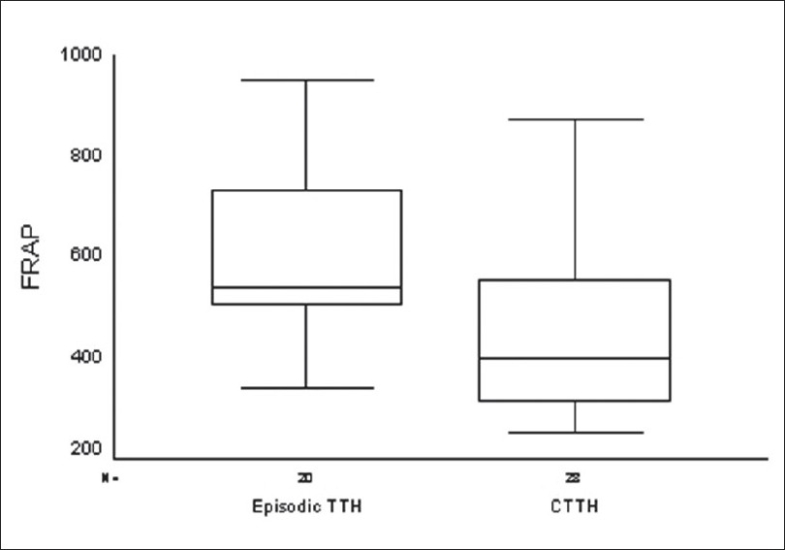
Comparison of plasma FRAP levels (μM) in the two TTH subgroups

**Figure 6:**
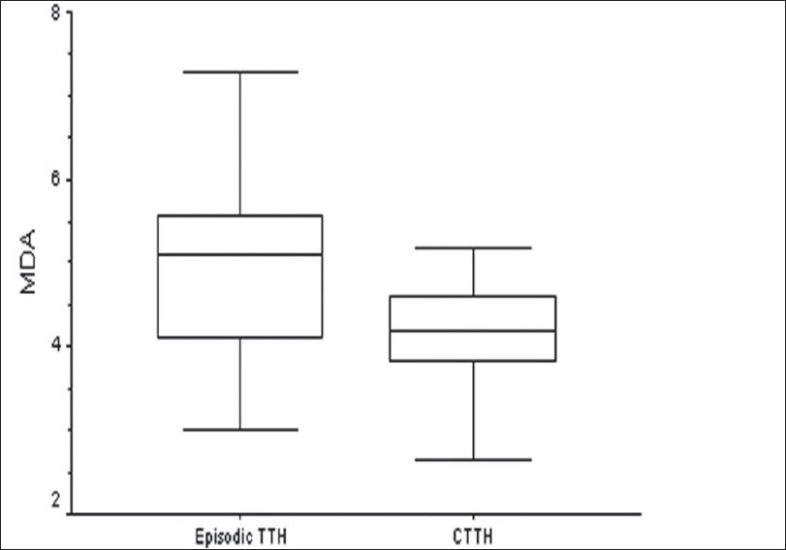
Comparison of plasma MDA levels (nmol/ml) in the two TTH subgroups

Similarly, the TTH group was also spilt into subgroups, i.e., ‘episodic TTH’ and ‘chronic TTH.’ We found that both FRAP (t = 3.16; P = 0.003) and MDA (t = 2.75; *P* = 0.008) levels were significantly different between these groups, with higher levels being seen in the ‘episodic TTH’ subgroup [Figures 5–6].

## Discussion

In this study oxidative stress was found to be higher among migraineurs as compared to the other two groups. This finding is in concordance with the previous findings, which show that migraineurs suffer from higher levels of oxidative stress than controls and those with TTH even in the inter-episodic period.[12,14,15] Shimomura *et al*,[12] found that the levels of oxidative stress were not different between the TTH and control groups (which is similar to the results of the present study) even when the blood sample was collected from TTH patients during the headache phase. These results suggest that although migraine is an episodic illness, the underlying biochemical changes leading to oxidative stress are long lasting or, possibly, even permanent after some time.

Increased MDA levels in migraineurs reflect the increased lipid peroxidation, while the higher levels of FRAP reveal the higher total antioxidant power in migraineurs as compared to the other two groups. Lipid peroxidation in migraineurs occurs secondary to the oxidative and nitrative stress. Migraineurs suffer from impaired brain energy phosphate metabolism during the initial stage of their attack.[1] However, the presence of oxidants in the inter-episodic period suggests that mitochondrial dysfunction in migraineurs is not episodic but extends also into the headache-free interval; this may destabilize the neurons and increase the propensity for having migraine.[20] The presence of mitochondrial dysfunction and disturbances in the electron transport system is further substantiated by the fact that the substances which enhance mitochondrial phosphorylation potential, e.g., riboflavin, coenzyme Q, niacin,[21] and thioctic acid, are beneficial for the prophylaxis of migraine.[22] Among these, riboflavin, niacin, and coenzyme Q increase the substrate availability to complex I of the mitochondrial respiratory chain.[21] The role played by mitochondria is further supported by the evidence of therapeutic benefit shown by carnitine in migraineurs. Deficiency of carnitine palmityl transferase-II (CPT-II) – an enzyme that is used for the transportation of fatty acids inside mitochondria – induces migraine, which improves with the administration of carnitine.[23] Furthermore, the blood levels of pyruvic acid and lactic acid are higher in migraineurs than in controls and TTH subjects, thus providing direct evidence of mitochondrial dysfunction in migraineurs.[24] Hence, there is enough evidence to support the theory that migraineurs have higher oxidant levels. However, mitochondrial dysfunction has never been reported in TTH and its presence is highly unlikely in the control group in this study. This explains the lower levels of oxidative stress seen in these groups.

Besides mitochondrial dysfunction, migraineurs also have higher levels of NO products in their blood during the inter-episodic period.[8,14] This can be related to the higher basal activity of the L-arginine/NO pathway, especially in patients of migraine with aura[25,26] and without aura.[25] These products react with superoxide to form peroxynitrite, which then induces lipid peroxidation and thus results in higher MDA levels in the migraineurs. FRAP is the biomarker for the total antioxidant activity of plasma and it should be decreased when the levels of MDA increase. However, in present study the opposite was found, and FRAP values were highest in migraineurs. This probably shows that as oxidative stress increases in the body, defence mechanisms come into play and increase the antioxidant power, till a critical limit is reached when the defences are exhausted. Further studies are required to address this issue and confirm it.

In the present study, oxidative stress was highest in subjects with migraine without aura and the levels were significantly different from that seen in subjects suffering from mixed migraine (episodes with and without aura); however, there was no significant difference from the level seen in the MA group. Shimomura *et al*,[12] showed that SOD concentration was higher in the MO group than in the MA group. A high activity of SOD is thought to represent better antioxidant power and this saves the body from oxidative-mediated damage.[27] Gallai *et al*.[26] provided evidence that the basal function of the L-arginine/NO pathway may be different in subjects with and without aura, with the subjects with aura having higher basal activity. NO levels are lower in MO subjects than in the MA subjects outside the headache period and, thus, the MO group has less chance of suffering nitrosative stress.[28] Together, these findings suggest that the MA group had higher chances of suffering from oxidative damage. On the contrary, Cinarcarelli *et al*.[14] did not find any difference between MO and MA subjects' urinary nitrate metabolite levels during the headache phase, after the headache, and during the headache-free period. Hence, it is difficult to conclude whether aura affects the levels of NO metabolites in the body. The different levels of NO and its metabolites reported by different studies could be due to differences in the methodologies used and also because NO can disappear quickly due to its highly reactive nature.[27] Moreover, the presence of aura may not affect the levels of thiobarbituric acid reactive substances (TBARS), suggesting that there could be some overlap of the pathological features of the subtypes of migraine or that inflammatory mechanisms are not involved in the development of aura at least.[14] Hence, this issue requires more detailed investigation, with better methodologies and larger samples.

Though the TTH group was not different from the control group, the levels were different between subjects with episodic TTH and chronic TTH. Moreover, as in migraineurs, TBARS and FRAP concentrations in this group were also minimally affected by disease-related variables. Similarly, at present we do not have concrete evidence that the biochemical changes in TTH are similar to that seen in migraine, particularly those changes that affect the oxidant status of a person. Hence, this area is also open for future research with improved methodology. The difference between the episodic TTH and chronic TTH groups could be due to development of protective mechanisms in the latter group.

The strength of this study lies in the strict exclusion criteria that we used. Firstly, none of the subjects included in this study had any other illness or confounding factor. Hence the findings in this study were exclusively the result of migraine and TTH. Secondly, most of the studies done in the past used clinical criteria for diagnosis of migraine and TTH, which leads to inclusion of non-identical subjects. We followed the ICHD-2 criteria[17] for diagnosis of migraine and TTH and hence we could avoid any selection bias.

However, this study also had some methodological limitations. First, the strict inclusion criteria that we used in this study precludes generalization of the results, as subjects with such isolated illness are not very common in the clinical population. Hence, results must be applied with caution in the general population. Second, owing to some technical factors, the sample size in this study was small relative to the prevalences of the illnesses in question. Third, the results of this study are based on only a cross-sectional examination during a specified period of the illness. Sequential measurements made at different phases of the illness would have been useful for elucidating the underlying pathophysiology in the subgroups included in this study. Fourth, the MDA is a relatively nonspecific marker for the measurement of lipid peroxidation. Despite that, measurement of MDA is one of the most popular methods owing to its simplicity and, hence, we chose this method. In future, studies can be planned using more specific measures.

In essence, this study suggests that the level of oxidative stress is higher even between headache episodes in migraineurs but not in those with TTH. This could probably be consequent to the different pathophysiological mechanisms of TTH and migraine.
